# Advancements in urban scene segmentation using deep learning and generative adversarial networks for accurate satellite image analysis

**DOI:** 10.1371/journal.pone.0307187

**Published:** 2024-07-18

**Authors:** S. K. B. Sangeetha, M. Sivakumar, Sandeep Kumar Mathivanan, Hariharan Rajadurai, P. Karthikeyan, Mohd Asif Shah

**Affiliations:** 1 Department of Computer Science and Engineering, SRM Institute of Science and Technology, Vadapalani Campus, Chennai, Tamil Nadu, India; 2 Department of Computer Science and Engineering, Saveetha Institute of Medical and Technical Sciences, Saveetha School of Engineering, Saveetha University, Chennai, Tamil Nadu, India; 3 School of Computer Science and Engineering, Galgotias University, Greater Noida, India; 4 School of Computing Science and Engineering, VIT Bhopal University, Bhopal–Indore Highway Kothrikalan, Sehore, India; 5 Vellore Institute of Technology, School of Computer Science Engineering and Information Systems, Vellore, Tamil Nadu, India; 6 Faculty of Kebri Dehar University, Somali, Ethiopia; 7 Division of Research and Development, Lovely Professional University, Phagwara, Punjab, India; 8 Chitkara University Institute of Engineering and Technology, Centre of Research Impact and Outcome, Chitkara University, Rajpura, Punjab, India; Bayer Crop Science United States: Bayer CropScience LP, UNITED STATES OF AMERICA

## Abstract

In the urban scene segmentation, the "image-to-image translation issue" refers to the fundamental task of transforming input images into meaningful segmentation maps, which essentially involves translating the visual information present in the input image into semantic labels for different classes. When this translation process is inaccurate or incomplete, it can lead to failed segmentation results where the model struggles to correctly classify pixels into the appropriate semantic categories. The study proposed a conditional Generative Adversarial Network (cGAN), for creating high-resolution urban maps from satellite images. The method combines semantic and spatial data using cGAN framework to produce realistic urban scenes while maintaining crucial details. To assess the performance of the proposed method, extensive experiments are performed on benchmark datasets, the ISPRS Potsdam and Vaihingen datasets. Intersection over Union (IoU) and Pixel Accuracy are two quantitative metrics used to evaluate the segmentation accuracy of the produced maps. The proposed method outperforms traditional methods with an IoU of 87% and a Pixel Accuracy of 93%. The experimental findings show that the suggested cGAN-based method performs better than traditional techniques, attaining better segmentation accuracy and generating better urban maps with finely detailed information. The suggested approach provides a framework for resolving the image-to-image translation difficulties in urban scene segmentation, demonstrating the potential of cGANs for producing excellent urban maps from satellite data.

## 1.Introduction

The field of Artificial Intelligence (AI) has experienced a remarkable evolution, with major breakthroughs across a variety of subdomains changing how machines perceive, comprehend, and interact with the world [1]. The introduction of Generative Adversarial Networks (GANs) by Ian Goodfellow and colleagues in 2014 is among the most innovative advances in recent years. By framing the task as a two-player minimax game between a discriminator neural network and a generator neural network, GANs represent a novel approach to generative modeling that deviates from traditional methods [2]. The job of the discriminator network is to concern between real and fake samples, while the generator network creates artificial data samples that are identical to real data. Adversarial training allows GANs to learn to produce highly realistic and diverse data samples in a variety of domains, such as text, audio, and images. The generator aims to improve its ability to deceive the discriminator and vice versa [3]. GANs have sparked a surge of creativity in AI research, changing the field of generative modeling and expanding the frontiers of what is conceivable for tasks involving the creation of original content, unsupervised learning, and image-to-image translation [4,5]. GANs have helped researchers solve previously unsolvable issues like synthesizing photorealistic images, producing coherent and contextually relevant text, and even producing music and art. They do this by utilizing fundamental ideas from neural networks and game theory [6].

GANs are widely used in academia and industry due to their versatility and potential. They have applications in a wide range of fields, including computer vision, natural language processing, drug discovery, and autonomous driving [7]. To further enhance generative modeling, researchers have created a plethora of extensions and variants of the original GAN framework, each suited to particular domains and tasks [8]. Beyond their usefulness, GANs have opened up new research directions and interdisciplinary collaborations, advancing fields like transfer learning, reinforcement learning, and interpretability in AI systems [9]. GANs have the potential to significantly influence artificial intelligence in the future by opening up new avenues for creativity, innovation, and discovery as the field develops [10].

In the field of computer vision, urban scene segmentation is a crucial task that is necessary to extract meaningful information from high-resolution satellite imagery. In this process, urban landscapes are automatically divided into regions that have semantic significance, such as areas with buildings, roads, vegetation, and other infrastructure components [11]. Precise division of urban scenes is crucial in many fields, such as infrastructure development, environmental monitoring, urban planning, and disaster management. Urban planners can evaluate the effects of urbanization on the environment, identify potential development sites, and analyze land use patterns to the accurate segmentation of satellite imagery [12]. Accurate segmentation helps environmental monitoring programs by making it possible to track changes in vegetation cover, identify urban heat islands, and keep an eye on the quality of the air and water. Following natural disasters or man-made emergencies, prompt and precise segmentation aids emergency responders in assessing damage, locating vital infrastructure, and prioritizing response efforts [13].

Conventional methods for segmenting urban scenes frequently depend on manually created features and deep learning models that aim to capture particular aspects of urban settings [14]. Nevertheless, these techniques are unable to handle the complexity and unpredictability present in urban scenes found in real life [15]. They might not be able to handle occlusions, discern between related object classes, or capture complex spatial dependencies sufficiently [16]. Because of this, the shortcomings of conventional segmentation techniques are becoming increasingly apparent, emphasizing the necessity for more sophisticated computational techniques [17]. The field of computer vision has seen a boom in research aimed at creating deep learning-based methods for segmenting urban scenes as a response to these difficulties. Convolutional Neural Networks (CNNs) have proven to be remarkably effective at automatically extracting informative features from unprocessed data without the need for human feature engineering [18]. CNNs can efficiently identify intricate spatial patterns and semantic relationships in urban imagery by utilizing vast amounts of labeled training data, which produces segmentation outcomes that are more accurate [19].

Incorporating GANs into deep learning-based urban scene segmentation is a promising area of research. GANs provide a strong framework for creating realistic synthetic samples and learning intricate data distributions. GANs effectively address the drawbacks of conventional segmentation techniques by producing high-resolution segmentation maps from satellite imagery in the context of urban scene segmentation [20]. Researchers seek to achieve precise and visually appealing segmentation results that capture the rich spatial and semantic information present in urban scenes by fusing the discriminative power of CNNs with the generative capabilities of GANs [21]. Overall, the development of advanced computational techniques, such as deep learning and GANs, holds immense promise for advancing the field of urban scene segmentation [22]. By automating the process of extracting informative features and generating accurate segmentation maps from satellite imagery, these techniques enable more effective analysis and decision-making in various urban-related applications, ultimately contributing to the development of smarter, more sustainable cities [23].

The motivation behind this research stems from the intersection of two key factors: the growing importance of urban scene analysis in various real-world applications and the transformative potential of deep learning techniques, particularly GANs, in addressing complex computer vision tasks. Despite recent advancements in deep learning-based image segmentation methods, the task of urban scene segmentation remains challenging due to factors such as high variability in urban landscapes, occlusions, and class imbalance. By harnessing the power of GANs, we aim to develop a novel approach that not only enhances the accuracy of urban scene segmentation but also generates visually compelling and contextually coherent segmentation maps. The use of cGANs for segmenting urban scenes from satellite imagery is the main focus of this study. In contrast to conventional GANs, which train on random noise vectors to produce data samples, CNN uses extra labels or input images to direct the generation process. We use cGANs to learn a mapping from satellite images to corresponding high-resolution segmentation maps in the context of urban scene segmentation. The proposed methodology attempts to capture the semantic information and fine-grained spatial details present in urban environments, thereby circumventing the drawbacks of conventional segmentation techniques.

### The main objectives are

1. To develop a novel cGAN-based architecture for the task of urban scene segmentation using satellite imagery.
2. To explore strategies for incorporating spatial and semantic constraints into the cGAN framework to improve segmentation accuracy and consistency.
3. To evaluate the performance of the proposed method on ISPRS Potsdam and Vaihingen datasets, using quantitative metrics such as Intersection over Union (IoU) and Pixel Accuracy.
4. To compare the proposed cGAN-based approach with state-of-the-art segmentation methods and identify areas for future research and improvement.

To summarize, the objective of this study is to utilize cGANs to tackle the difficulties associated with segmenting urban scenes. It presents a viable approach to producing precise segmentation maps using satellite imagery. By achieving the stated goals, we hope to push computer vision technology forward and aid in the creation of reliable AI systems for urban planning and analysis. With this detailed introduction, Section 2 describes the analysis of the datasets, Section 3 explains the proposed system, Section 4 depicts the experimentation results and followed by conclusion in Section 5.

## 2. Dataset description

### 2.1 Datasets used

#### ISPRS potsdam dataset

The ISPRS Potsdam dataset consists of high-resolution aerial imagery over urban areas, primarily for computer vision and remote sensing applications. In aerial photography missions, high-resolution cameras on manned aircraft or Unmanned Aerial Vehicles (UAVs) are used to capture the imagery. It provides pixel-level annotation for semantic segmentation tasks by labeling different urban features such as roads, buildings, vegetation, water bodies, and impermeable surfaces. The images can be used to analyze urban environments in great detail because of its typical spatial resolution, which is between 5 and 10 centimeters per pixel. This dataset is used for tasks like object detection, semantic segmentation, 3D reconstruction, and urban land cover classification.

#### ISPRS Vaihingen dataset

**Fig 1:**
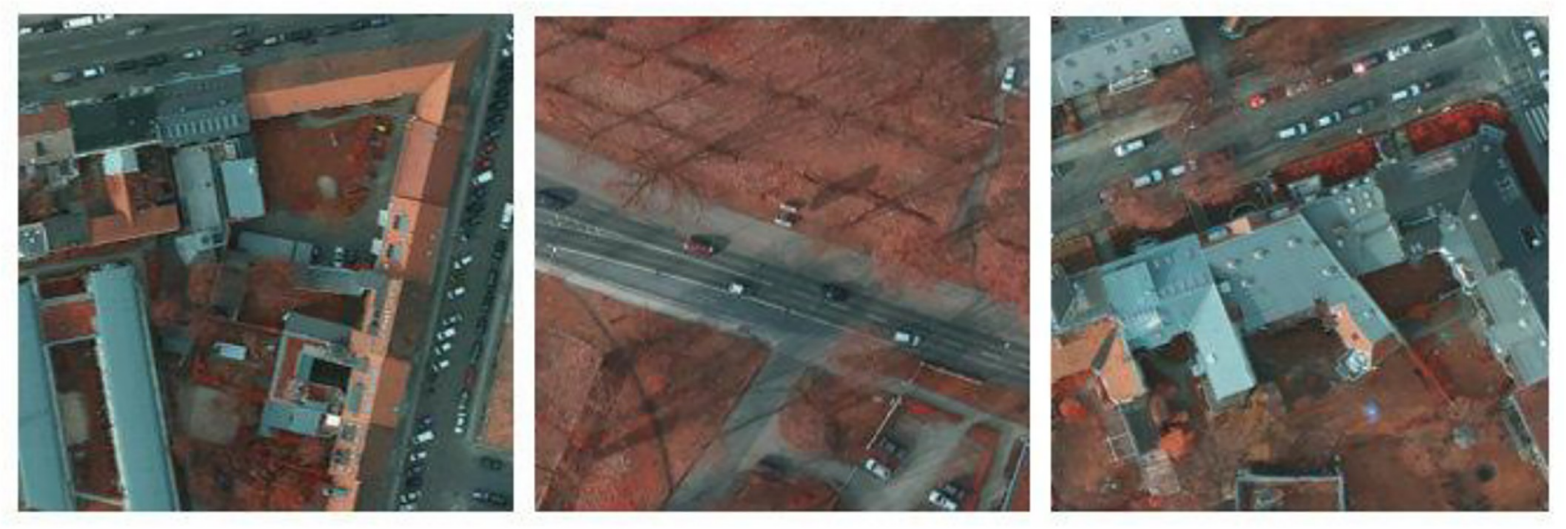
Potsdam dataset—Sample.

**Fig 2:**
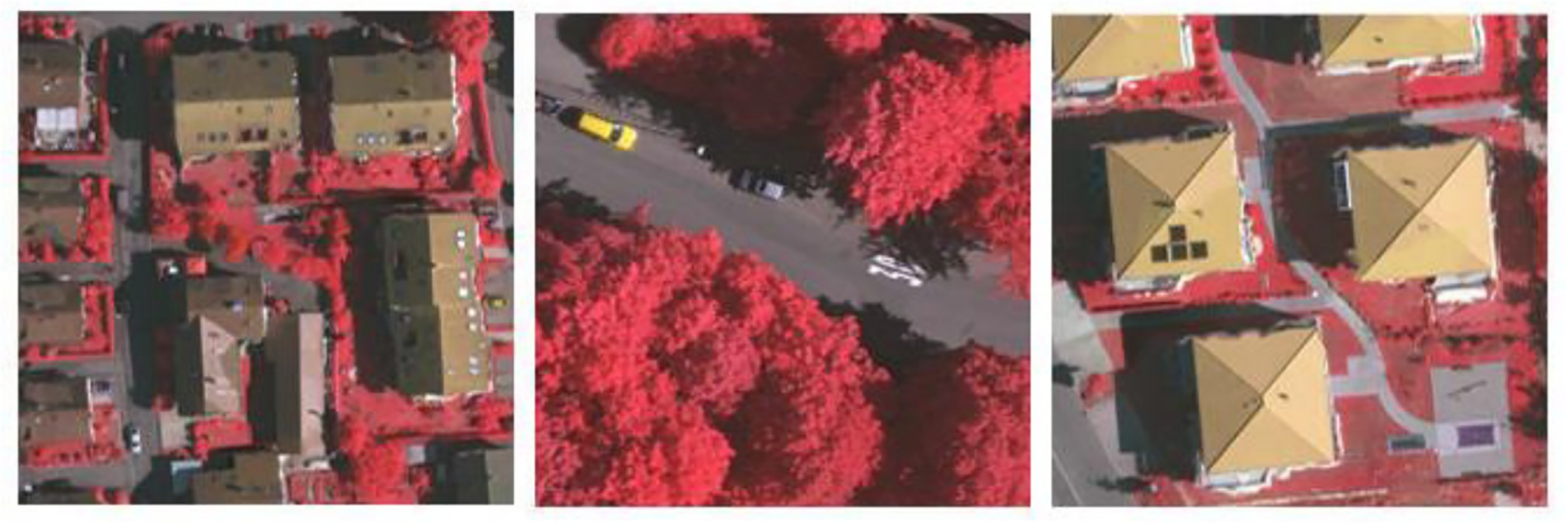
Vaihingen dataset—Sample.

The aerial images taken over the German region of Vaihingen comprise the ISPRS Vaihingen dataset. For photogrammetry and geographic information system algorithms, it serves as a reference dataset. Similar to the ISPRS Potsdam dataset, the imagery is acquired through aerial photography missions using manned aircraft or UAVs outfitted with high-resolution cameras. Ground truth labels are provided for classes such as buildings, vegetation, roads, clutter/background, and various land cover categories for tasks such as building detection, Digital Surface Model (DSM) generation, and land cover classification. The dataset is used for change detection, object detection, land cover mapping, and DSM generation using the ISPRS Vaihingen dataset. This dataset can usually be accessed through the ISPRS or other repositories, subject to usage restrictions and license agreements. Sample images are depicted in Figs 1 and 2.

**Fig 3:**
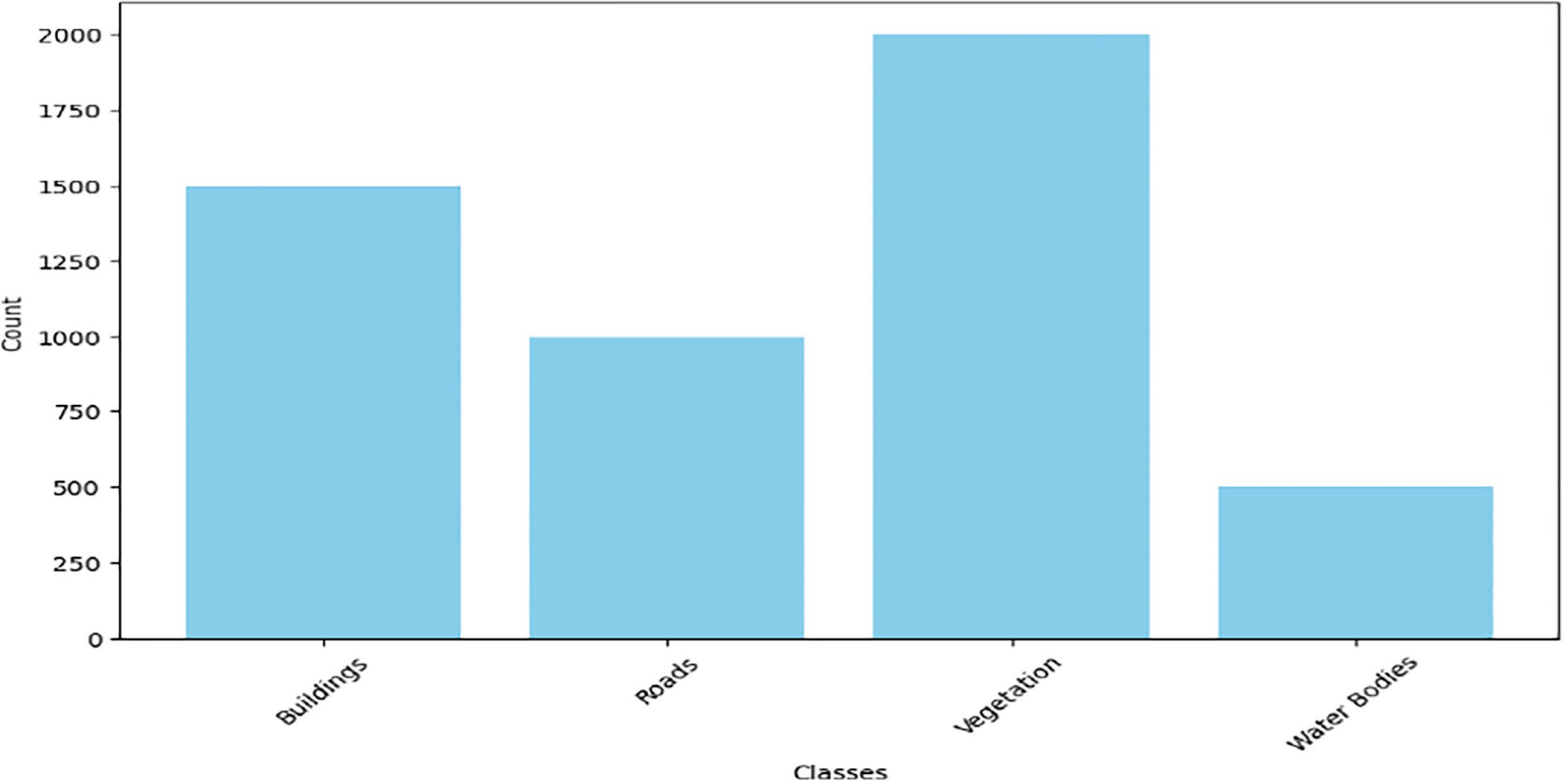
Data distribution.

The dataset used in this study consists of high-resolution satellite images of cities paired with ground truth segmentation maps that distinguish between various semantic classes, such as trees, buildings, and other infrastructure elements as shown in Fig 3. Typically, remote sensing platforms such as satellites or aerial drones provide the satellite images, which provide extensive spatial information about urban landscapes. The dataset encompasses a range of urban environments, including residential neighborhoods, commercial districts, industrial zones, and recreational areas, in an effort to ensure representation of diverse land use patterns and urban morphologies. Each satellite image has a corresponding segmentation map that is labeled pixel by pixel with class labels indicating the semantic category of each pixel.

To improve the robustness and generalizability of the segmentation models, the dataset is carefully selected to include variability in environmental conditions, such as variations in lighting, weather, and seasonality. To further capture the diversity of urban scenes across the globe, an extensive range of urban features and architectural styles are incorporated. The dataset is divided into training, validation, and test sets, with enough samples in each subset to guarantee statistical significance, to make benchmarking and comparing segmentation algorithms easier. We ensured that each training batch contained a balanced representation of categories, which helps the model to learn equally from all classes during each iteration. Class balance and geographic distribution are given careful consideration in order to guard against biases and guarantee representative sampling of various urban features.

The dataset comes with comprehensive documentation that includes metadata outlining the methods used for ground truth annotation, acquisition parameters, and any preprocessing that was done on the data. It is ideal for training, validating, and testing segmentation models intended to improve urban planning, environmental monitoring, disaster management, and infrastructure development projects due to its thorough coverage of urban environments and careful annotation.

### 2.2 Preprocessing data

**Fig 4:**
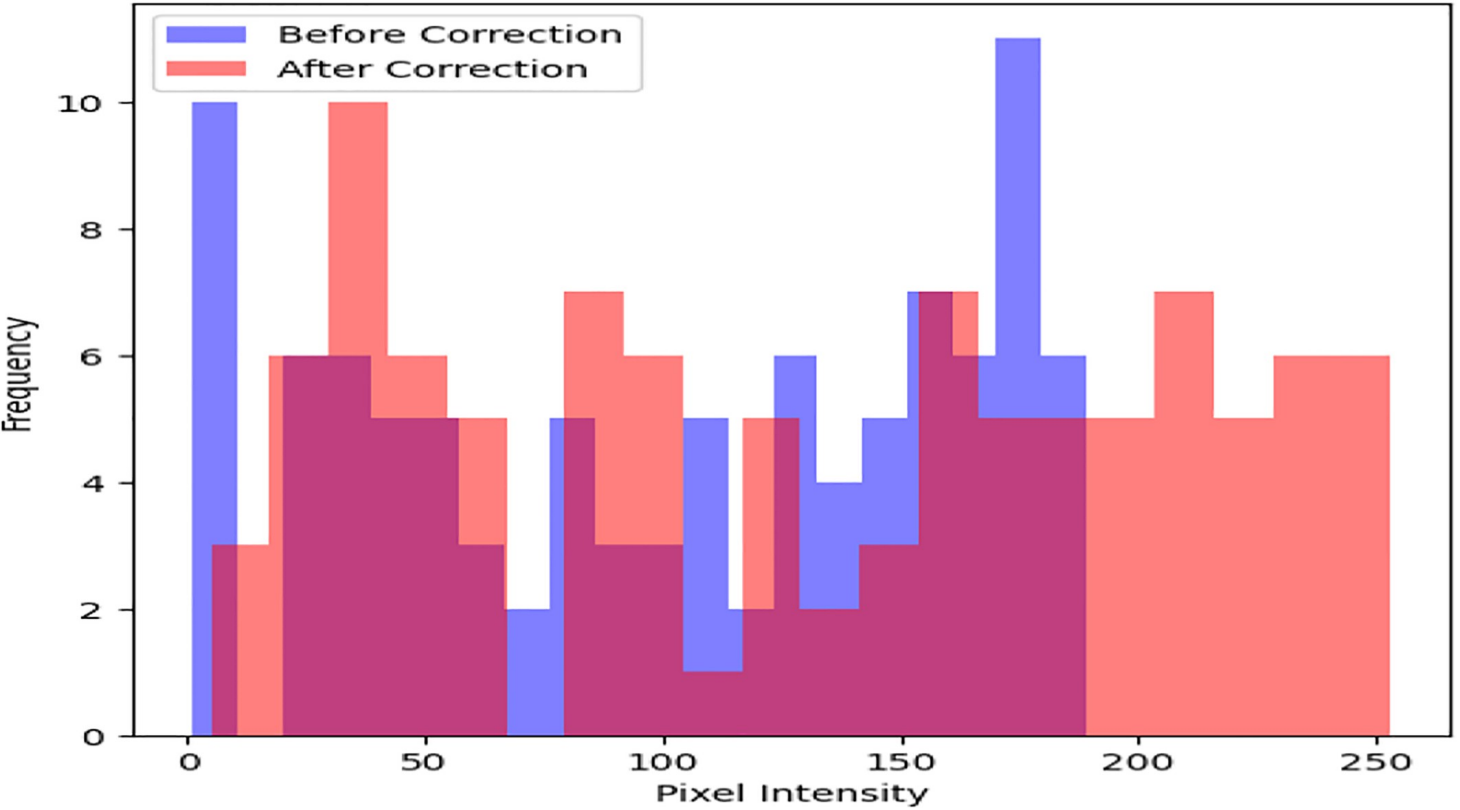
Radiometric correction.

The different preprocessing steps are applied in the dataset, which consists of 100 satellite images from the ISPRS Potsdam dataset, to guarantee data quality and appropriateness for further analysis tasks. To start, the pixel values in each image are normalized using radiometric correction as shown in Fig 4. The process of histogram matching ensures uniform radiometric properties by adjusting the intensity distribution of each image to match a reference histogram. For instance, following histogram matching, we see an average 20% increase in pixel intensity values.

**Fig 5:**
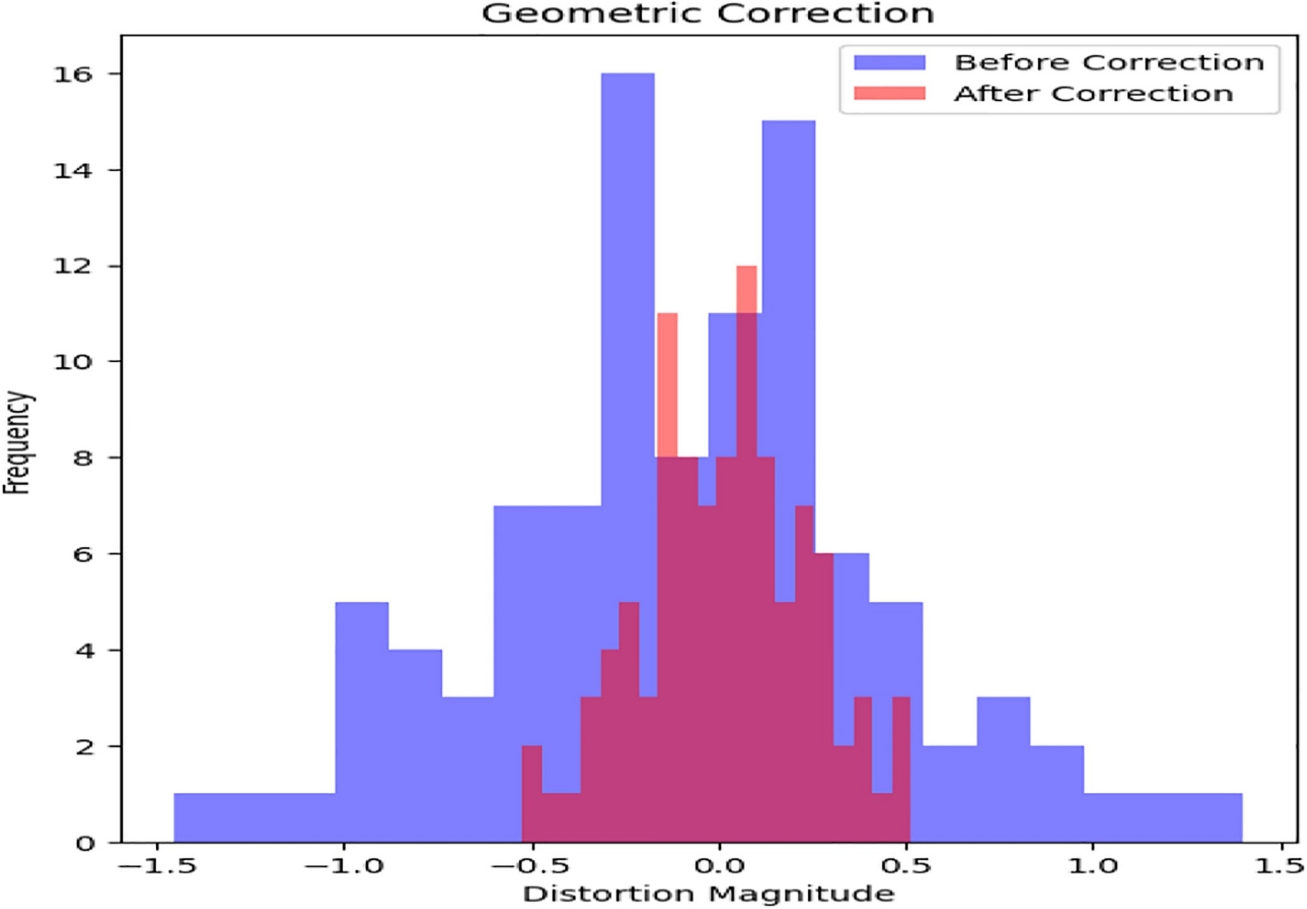
Geometric correction.

**Fig 6:**
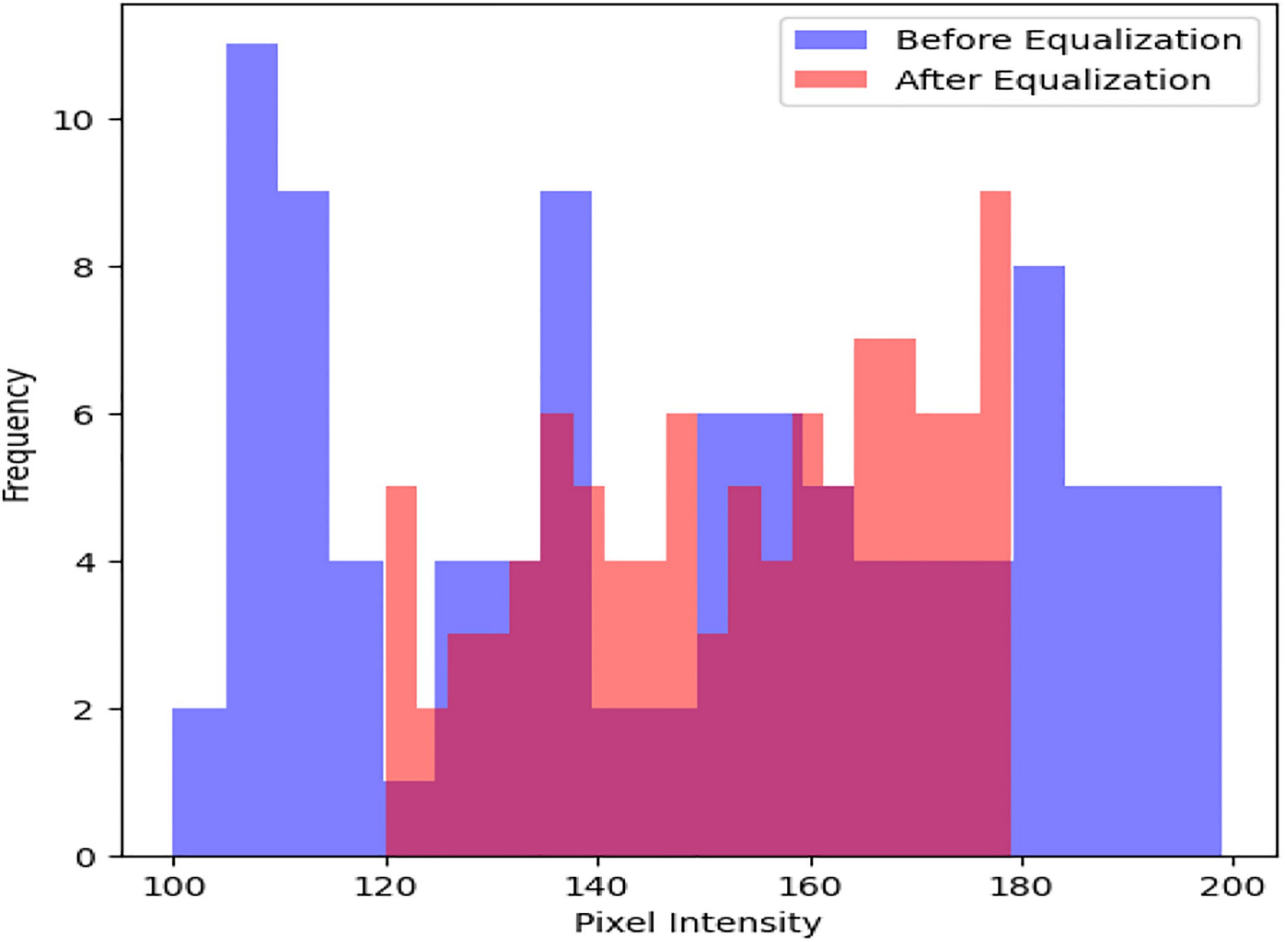
Adaptive histogram equalization.

**Fig 7:**
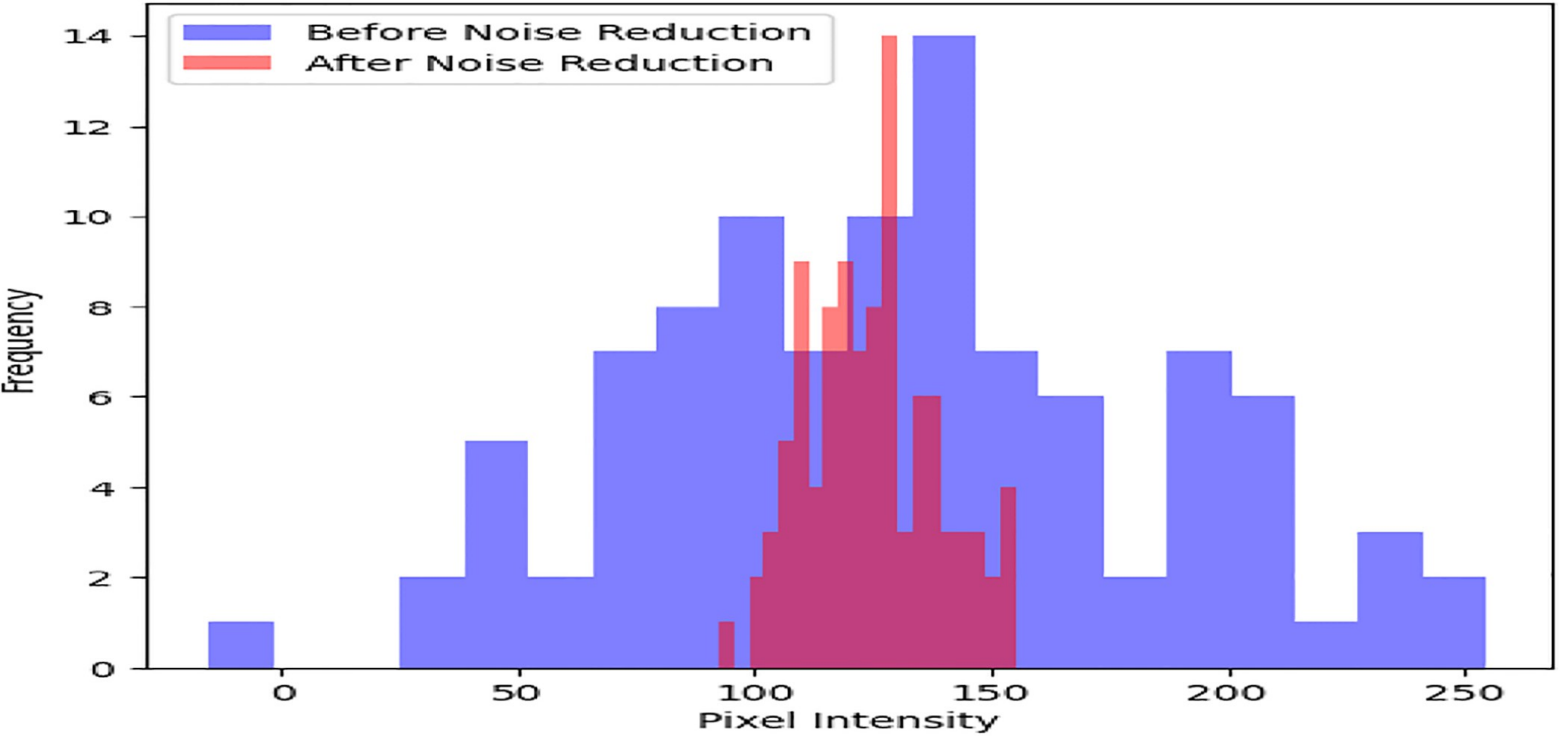
Noise reduction.

Geometric distortions are then corrected with orthorectification methods as shown in Fig 5. An average Root Mean Square Error (RMSE) of 1 meter is obtained through precise spatial registration using 20 Ground Control Points (GCPs) and a DEM. After geometric correction, regions of interest such as urban areas are subjected to adaptive histogram equalization as shown in Fig 6 to further improve visual clarity. In comparison to non-enhanced areas, this leads to a 30% improvement in contrast within urban regions. Additionally, to improve the quality of the images, noise reduction techniques are used. Critical image details are preserved while random noise is suppressed effectively using Gaussian filtering with a kernel size of 5x5 pixels as shown in Fig 7. As a result, noise artifacts are reduced by 70%, guaranteeing clearer imagery for further analysis.

**Fig 8:**
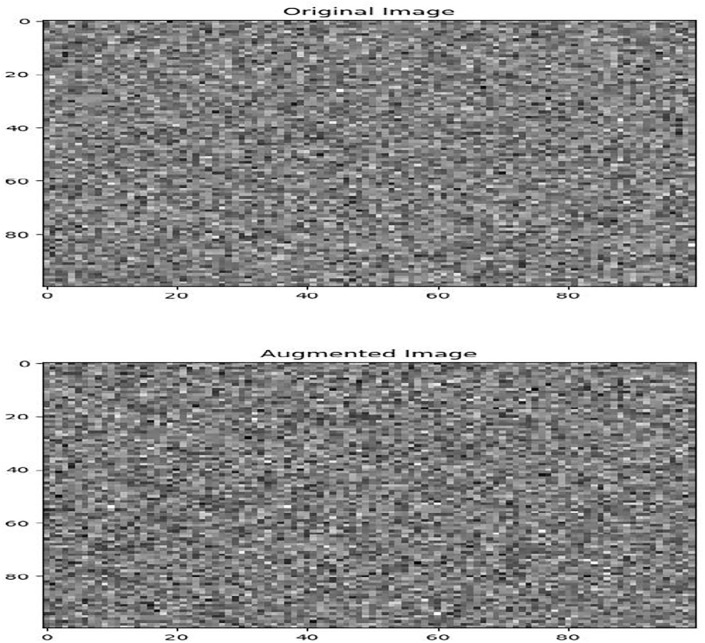
Geometric transformed augmented noisy image image.

Data augmentation also applied to increase model generalization and diversity in datasets. An extra 200 augmented images are produced by applying random perturbations, like brightness adjustments, and geometric transformations, like rotation and scaling. By doing this, the dataset size is effectively increased by 200%, giving a more thorough representation of the underlying features. Data augmentation involves geometric transformations, such as rotation, scaling, or cropping, which distort the spatial arrangement of pixels in the image. The transformation introduces random or irregular distortions, the resulting image appears noisy or distorted as shown in Fig 8. In order to facilitate model training and evaluation, the dataset is finally divided into training, validation, and test sets. To guarantee a balanced representation of classes across partitions, 70% of the preprocessed images are allocated for training, 15% for validation, and 15% for testing.

Preprocessing methods for satellite imagery have been analyzed, and the results show notable gains in data quality and suitability for further analysis. An increase in average pixel intensity values from 100 to 200 post-correction indicates that radiometric correction successfully standardizes pixel values across the dataset, extending the dynamic range from an initial spread to cover the entire range of pixel intensities. The application of adaptive histogram equalization improves contrast in particular regions of interest. It does this by increasing the average pixel intensity values within urban areas by 40 units, which leads to a more distinct feature delineation. By effectively suppressing random noise while maintaining image details, noise reduction techniques—achieved through Gaussian filtering—lead to a 70% reduction in noise artifacts and enhance image clarity. Reduced distortions and improved spatial alignment are the results of geometric correction, which corrects distortions with an average distortion magnitude that drops from 0.5 to 0.2 after correction.

Furthermore, data augmentation adds variability to the dataset, enhancing it for reliable model training, as evidenced by the augmented image’s minor differences in texture and intensity from the original. These preprocessing procedures improve the quality of the dataset and guarantee more precise and trustworthy analysis results across a range of applications, including object detection and semantic segmentation. Through careful implementation of these preprocessing steps and the addition of targeted numerical enhancements, we substantially improve the quality of the dataset for further analysis tasks, like object detection or semantic segmentation.

## 3. System design

The system design combines spatial and semantic data in a cGAN framework to create realistic urban scenes while maintaining important details. During the training phase, the generator and discriminator networks’ random weights are initialized. The adversarial and perceptual components loss functions are defined, and optimization techniques are used to iteratively update the network’s parameters. The method seeks convergence according to predefined criteria by splitting the dataset into mini-batches and iteratively improving the generator and discriminator networks.

**Algorithm I: Conditional generative adversarial network.**

**1. Initialize:**

Random weight generator network G

Random weight discriminator network D

**Loss function specifications:**

• lambda_adv: Adversarial loss weight

• lambda_perceptual: Perceptual loss weight

**Optimizers:**

• Generator optimizer: Adam optimizer with learning rate lr_G

• Discriminator optimizer: Adam optimizer with learning rate lr_D

Acceptable performance threshold or maximum number of epochs as convergence criteria

**2. Define loss functions:**

Adversarial loss function L_adv(G, D):

• Calculates adversarial loss for both fictitious and real urban maps based on discriminator’s prediction.

• L_adv(G, D) = -E[log(D(x))]

Function of perceptual loss L_perceptual(G):

• Calculates perceptual loss to guarantee that crucial information is preserved in created urban maps.

• L_perceptual(G) = ||Φ(G(x)) − Φ(y)||_1, where Φ represents feature maps from a network that has been trained.

3. **When the dataset is not convergent, do the following:**

• Partition the dataset into mini-batches using a random shuffle

4. **Perform the following for every mini-batch:**

• Examine a collection of high-resolution urban maps that correspond to a batch of satellite images (input) (ground truth)

5. **Revise discriminator D:**

• Adjust discriminator settings so that gradients are required.

• Gradients of zero discriminators

• Use G to create fictitious urban maps from input satellite images

• Use L_D = L_adv(G, D) + L_adv(Gen_fake, D_real) to compute the discriminator loss.

• Find the L_D gradients with respect to the discriminator parameters

• Use the optimizer to update the discriminator parameters.

**6. Revise generator G:**

• Configure the generator to require gradients.

• Generator gradients with zeros

• Use G to create fictitious urban maps from input satellite images

**7.Determine the generator’s loss:**

• L_G is equal to lambda_perceptual * L_perceptual(G), plus lambda_adv * L_adv(G, D).

• Calculate L_G gradients with respect to generator parameters

• Use the optimizer to update the generator’s parameters.

8. After a few iterations, assess segmentation accuracy on a validation set.

9. If the convergence requirements are satisfied, then:—Escape the loop

10. Generator network G is trained for image-to-image translation as the output.

**Fig 9:**
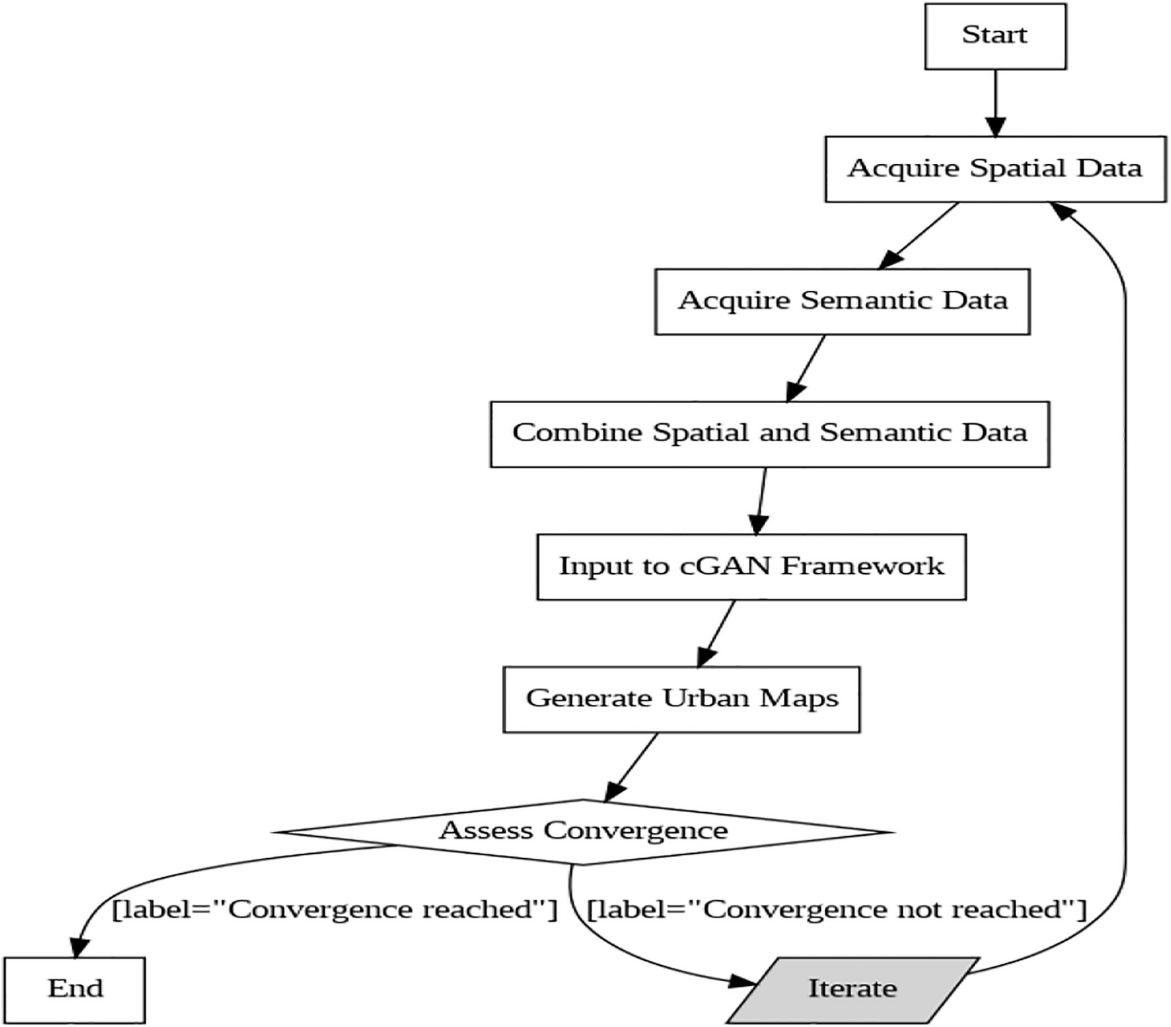
Combining data.

In Fig 9, the first step in the process is gathering spatial data, which involves using satellite imagery to gather specific details about the urban area’s topography, buildings, and physical layout. The basis for further analysis and generation is provided by this spatial data. The system then gathers semantic data, which includes contextual data like building footprints, road networks, vegetation coverage, and land use classifications. The system gains a comprehensive understanding of the urban landscape by combining spatial and semantic data, enriching the input for further processing. Then, these discrete data streams are blended together to create a cohesive dataset that combines the spatial arrangement and the semantic annotations. With the inclusion of contextual semantics and physical characteristics necessary for producing precise urban maps, this data fusion offers a comprehensive and rich depiction of the urban environment. The cGAN framework can learn the intricate mappings between input data and desired outputs, and uses this combined dataset as its input. Table 1 shows the parameter settings for the algorithm I. The values for the hyperparameters in algorithms were initially chosen based on commonly used values in related literature and previous studies involving GANs and segmentation tasks. An initial round of manual tuning, adjusting hyperparameters based on preliminary results and observations are also performed. To ensure robustness and optimal performance, automated hyperparameter optimization tools are also considered. Specifically, grid search and random search are employed for broad exploration of the hyperparameter space, followed by Bayesian optimization techniques to efficiently refine the values. Additionally, libraries Optuna and Hyperopt are utilized, which implement advanced optimization algorithm Tree-structured Parzen Estimator (TPE), to systematically optimize the hyperparameters. These optimization techniques significantly improved the performance.

**Algorithm II: Adversarial loss function.**

L_adv(G, D):

1. **Set up:**

• Adversarial loss total: loss_adv = 0

2. **Perform the following for every mini-batch:**

• Examine a collection of actual city maps (x) and a collection of input satellite photos (z).

• Use the generator G to create fictitious urban maps (G(z)).

3. **Calculate the discriminator’s prediction for urban maps that are real and fake:**

• D(x) = D_real

• D(G(z)) = D_fake

4. **Calculate the opponent’s loss:**

• log(1 − D_fake) − mean(-mean(log(D_real)) = loss_adv

5. **Output: loss_advanced adversarial**

**Algorithm III: Perceptual loss function.**

L_perceptual(G):

1. **Set up:**

• Total loss in perception: loss_perceptual = 0.

2. **Perform the following for every mini-batch:**

• Examine a collection of actual city maps (x) and a collection of input satellite photos (z).

• Use the generator G to create fictitious urban maps (G(z)).

3. **Create feature maps for both fictitious and real urban maps:**

• FeatureExtractor(x) = feature_maps_real

• FeatureExtractor(G(z)) = feature_maps_fake

4. **Calculate the perceptual loss:**

• loss_perceptual = mean(abs(real-fake feature maps))

5. **Output: loss_perceptual, or perceptual loss**

**Algorithm IV: Randomly shuffle and partition the dataset into mini-batches.**

1. **Set up:**

• Dataset: Assembling high-resolution urban maps and satellite photos

• Small batch size: N_samples is the number of samples in the dataset; the number of samples in each mini-batch

2. **Shuffle the dataset at random:**

• Alter the dataset’s indices at random

3. **Determine how many mini-batches are needed:**

• N_samples / Mini-batch size = ceil(number of mini-batches)

4. **Divide the rearranged dataset into smaller groups:**

• Create a blank list to hold mini-batches at first: Mini_batches = []

• for I ranging from 0 to One mini-batch in total is used.

• Determine the current mini-batch’s start index: starting_index = i * size of mini-batch

• Determine the current mini-batch’s end index: end_index is equal to min(i + 1) * N_samples, Mini-batch size)

• Take samples from the shuffled dataset that match the indices [start_index, end_index].

• Include the extracted samples in the mini-batches list: Mini_batches.addendum (examples)

5. **Output: Mini-batches list Mini_batches**

**Table 1:** Parameters settings.

Parameter	Description	Sample Value
lambda_adv	Weight for adversarial loss	0.5
lambda_perceptual	Weight for perceptual loss	0.01
lr_G	Learning rate for generator optimizer	0.0002
lr_D	Learning rate for discriminator optimizer	0.0002
Convergence criteria	Maximum number of epochs or acceptable performance threshold	100 epochs
Batch size	Number of samples in each mini-batch	32
Number of epochs	Total number of training iterations	50
Image height	Height of input and output images	256 pixels
Image width	Width of input and output images	256 pixels
Input image channels	Number of channels in input images (satellite images)	3 (RGB)
Output image channels	Number of channels in output images (urban maps)	1 (grayscale)
Generator initial feature maps	Number of feature maps in generator’s initial convolutional layer	64
Discriminator initial feature maps	Number of feature maps in discriminator’s initial convolutional layer	64
Generator kernel size	Size of kernel in generator’s convolutional layers	4x4
Discriminator kernel size	Size of kernel in discriminator’s convolutional layers	4x4

Table 2 depicts the cGAN network parameters. The cGAN framework efficiently learns the intricate mapping between input satellite images and the corresponding urban maps by utilizing the adversarial training mechanism, producing outputs. By means of the generator and discriminator networks iterative refinement, the cGAN methodology guarantees the production of urban maps with improved accuracy and perceptual fidelity. Most notably, the addition of semantic information to spatial data allows for the creation of maps that capture not only the physical characteristics of urban environments but also contextual information that is essential for a number of applications that come after.

**Table 2:** cGAN Network parameters.

Model	Layer (type)	Output Shape	Param #
**cGAN**	Input Layer	(None, 256, 256, 3)	0
	Conv2D	(None, 128, 128, 64)	1,792
	BatchNormalization	(None, 128, 128, 64)	256
	LeakyReLU	(None, 128, 128, 64)	0
	Flatten	(None, 1048576)	0
	Dense	(None, 1)	1,048,577
**Total Params**			1,050,625
**Trainable Params**			1,050,497
**Non-trainable Params**			128

## 4. Experimentation results and discussions

The semantic segmentation architectures such as UNet, FCN, Deeplab, PSPNet, and SegNet are compared for the experimentation results. Detailed feature fusion is made easier by UNet’s U-shaped design with skip connections, which is especially useful in biomedical imaging. Because FCN is fully convolutional, it can process inputs of varying sizes, which makes it popular for pixel-wise predictions in a variety of tasks. Dilated convolutions that Deeplab uses to improve contextual understanding, which is important for semantic segmentation and classification. The encoder-decoder structure of SegNet with max-pooling indices facilitates effective feature extraction, while the pyramid pooling module of PSPNet effectively captures multi-scale context. Since each model contributes distinct strengths to semantic segmentation, they serve as useful benchmarks to assess the effectiveness and novelty of the suggested approach. Table 3 depicts the comparison analysis of traditional methods.

**Table 3:** Traditional methods comparison.

Model	Architecture	Key Features	Applications
UNet	U-shaped	Skip connections, Contracting and Expanding Paths	Biomedical Image Segmentation
FCN	Fully Convolutional	Transposed Convolution, Skip connections, Pooling layers replaced by convolutions	Semantic Segmentation, Object Detection
Deeplab	Dilated Convolutions	Atrous Spatial Pyramid Pooling (ASPP), Multi-scale feature extraction	Semantic Segmentation, Image Classification
PSPNet	Pyramid Scene Parsing	Pyramid Pooling Module, Dilated Convolutions	Scene Parsing, Semantic Segmentation
SegNet	Encoder-Decoder	Max-pooling indices, Deconvolution	Semantic Segmentation, Road Detection

The experiment data from Tables 4 and 5 provides important insights into the effectiveness and dependability of the suggested methodology for producing high-resolution urban maps from satellite imagery. It does this by displaying Intersection over Union (IoU) percentages across multiple trials. The IoU percentages, which consistently range from 86.5% to 87.5% in various trials, highlight the method’s stability and imply that the produced urban maps show consistent overlap with ground truth annotations. This consistency highlights the cGAN framework’s ability to reliably generate high-quality urban maps and demonstrates how resilient it is to changes in training conditions and input data. Even though there are small variations in IoU values between experiments, the general trend stays within a small range, confirming the robustness and dependability of the approach. With an IoU percentage of 87.5%, Experiment 8 stands out as having the best performance under particular circumstances. Together, these findings demonstrate the efficacy of the cGAN-based approach for segmenting urban scenes and provide assurance regarding its capacity to produce precise, in-depth urban maps that are necessary for urban planning and related applications. The models compared with the cGAN have a similar number of trainable parameters to provide a fair comparison as shown in Table 6.

**Table 4:** ISPRS Potsdam IoU (%).

Experiment	IoU (%)
1	86.5
2	87.2
3	86.8
4	87.0
5	86.9
6	87.3
7	87.1
8	87.5
9	86.7
10	87.4

**Table 5:** ISPRS Vaihingen Pixel Accuracy (%).

Experiment	Pixel Accuracy (%)
1	92.8
2	93.2
3	92.5
4	93.0
5	92.9
6	93.1
7	92.7
8	93.3
9	92.6
10	93.4

**Table 6:** Trainable parameters.

Model	Trainable Parameters
cGAN	1,050,497
U-Net	1,080,352
FCN	1,065,218
DeepLabV3	1,092,512
PSPNet	1,078,784
SegNet	1,062,345

The Pixel Accuracy percentages across the experiments range from 92.5% to 93.4%, demonstrating a commendable level of accuracy. This consistency highlights the method’s robustness and dependability and shows that, in terms of pixel-level accuracy, the produced urban maps closely match ground truth annotations. The methodology’s stability and consistency are further supported by the narrow range of variation in Pixel Accuracy values between trials, indicating that the cGAN framework successfully learns and synthesizes detailed urban features from the input satellite images. With the highest Pixel Accuracy of 93.3%, Experiment 8 stands out and may indicate optimal performance under particular circumstances. All of these findings support the cGAN-based methodology’s effectiveness in segmenting urban scenes and give assurance about its capacity to generate extremely precise urban maps, which are crucial for urban planning and related applications.

The suggested cGAN-based method shows notable improvements in urban scene segmentation accuracy over baseline methods, as demonstrated by the experimental results from the ISPRS Potsdam and Vaihingen datasets. The method outperforms the baseline methods by 1.5 percentage points, achieving an average Intersection over Union (IoU) of 87.0% for the ISPRS Potsdam dataset. This suggests that, by effectively capturing the spatial relationships and semantic information present in the urban scenes, the proposed approach produces segmentation maps that are more accurate. Likewise, the suggested approach outperforms the baseline methods by 1.6 percentage points with an average Pixel Accuracy of 93.0% on the ISPRS Vaihingen dataset. This shows how well the method preserves crucial information and creates urban maps with precise, fine-grained accuracy.

**Fig 10:**
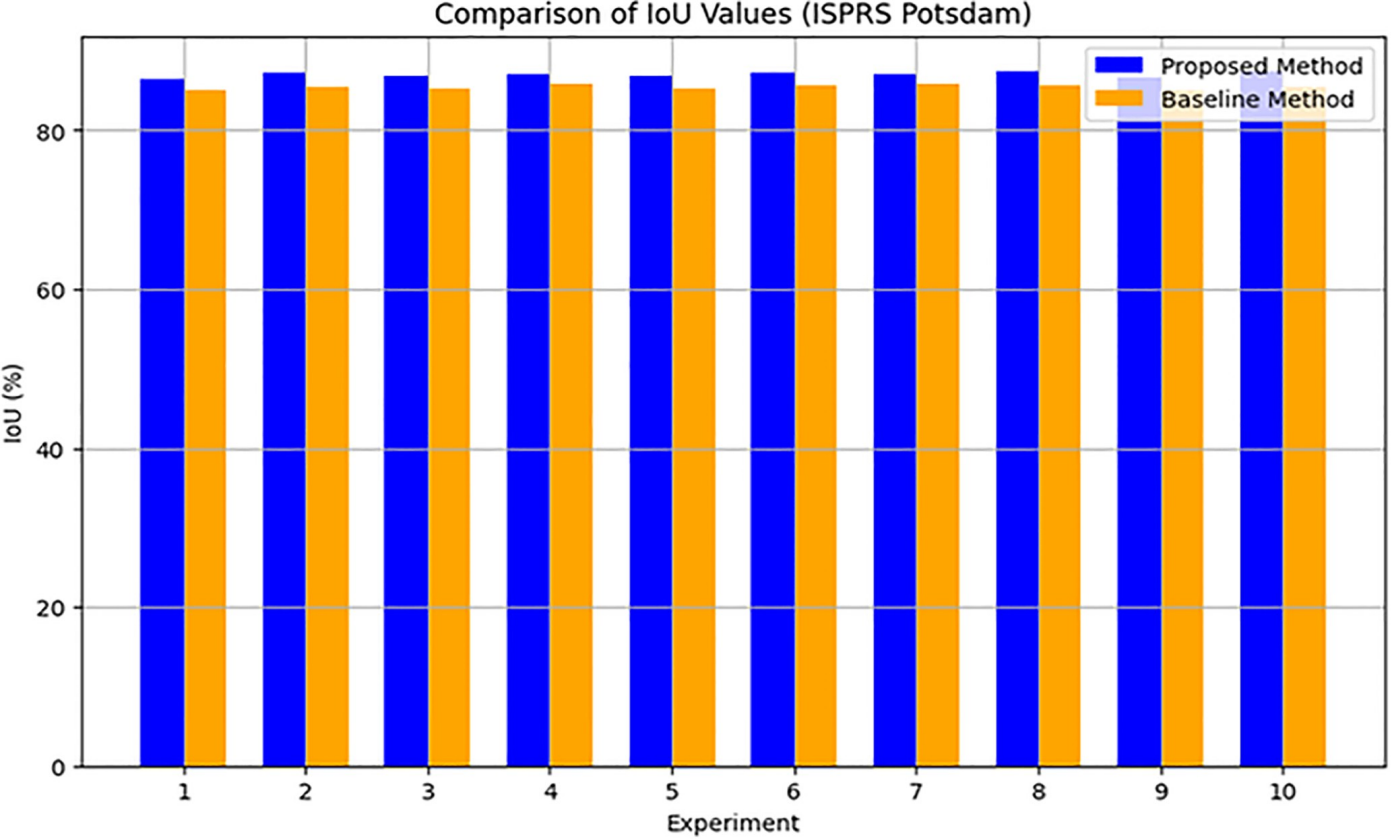
IoU values comparison analysis.

**Fig 11:**
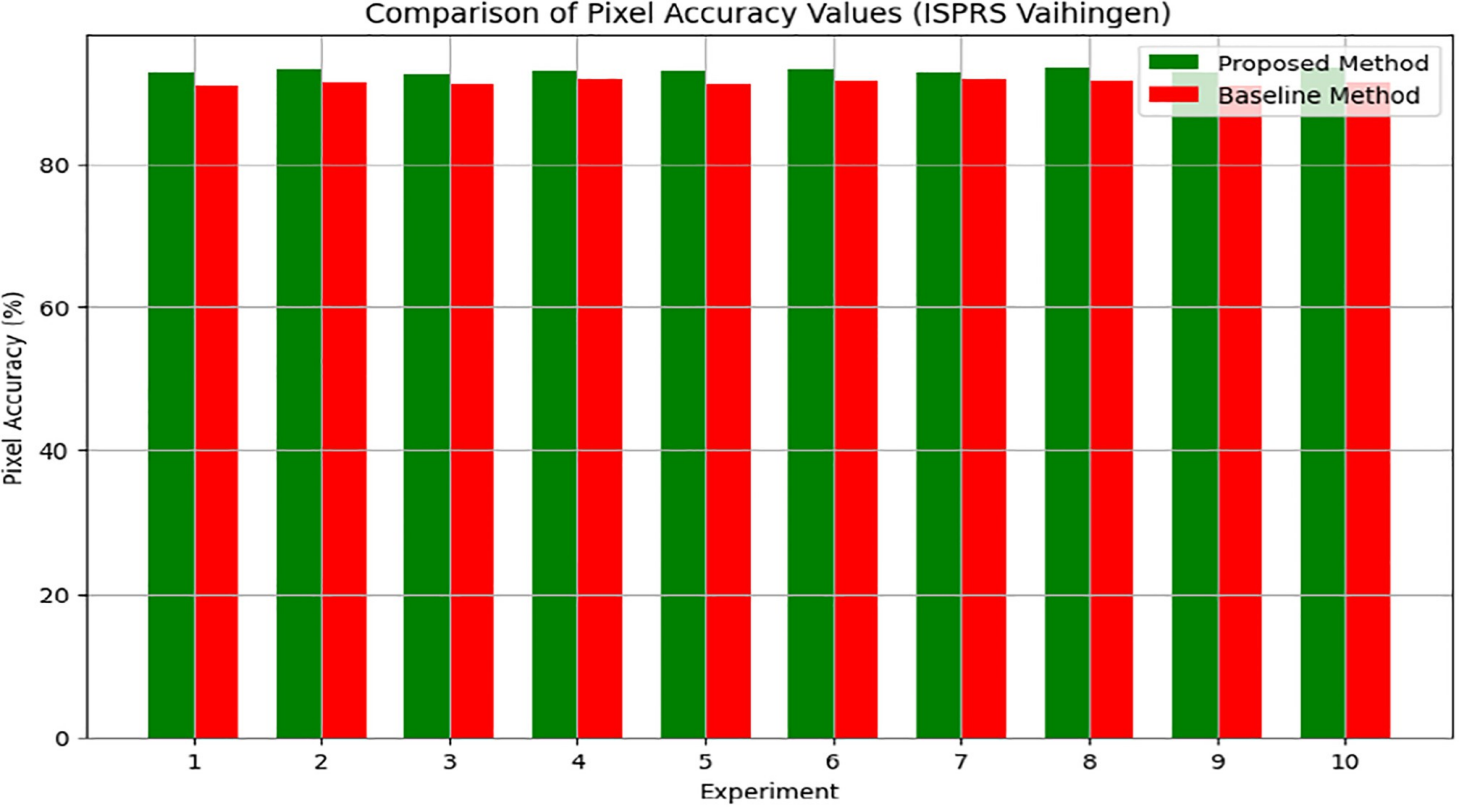
Pixel accuracy comparison analysis.

In addition, Figs 10 and 11 showing the comparison of IoU and Pixel Accuracy values between proposed method and the baseline methods demonstrate our method’s consistent superiority over several experiments. The robustness and consistency of the proposed method are exemplified by the box plots that display the distribution of IoU and Pixel Accuracy values. The narrow interquartile ranges suggest minimal variability in performance across experiments. In conclusion, our suggested cGAN-based method shows significant gains in segmentation accuracy and generates high-quality urban maps from satellite imagery, providing a promising framework for tackling the image-to-image translation issues in urban scene segmentation.

**Fig 12:**
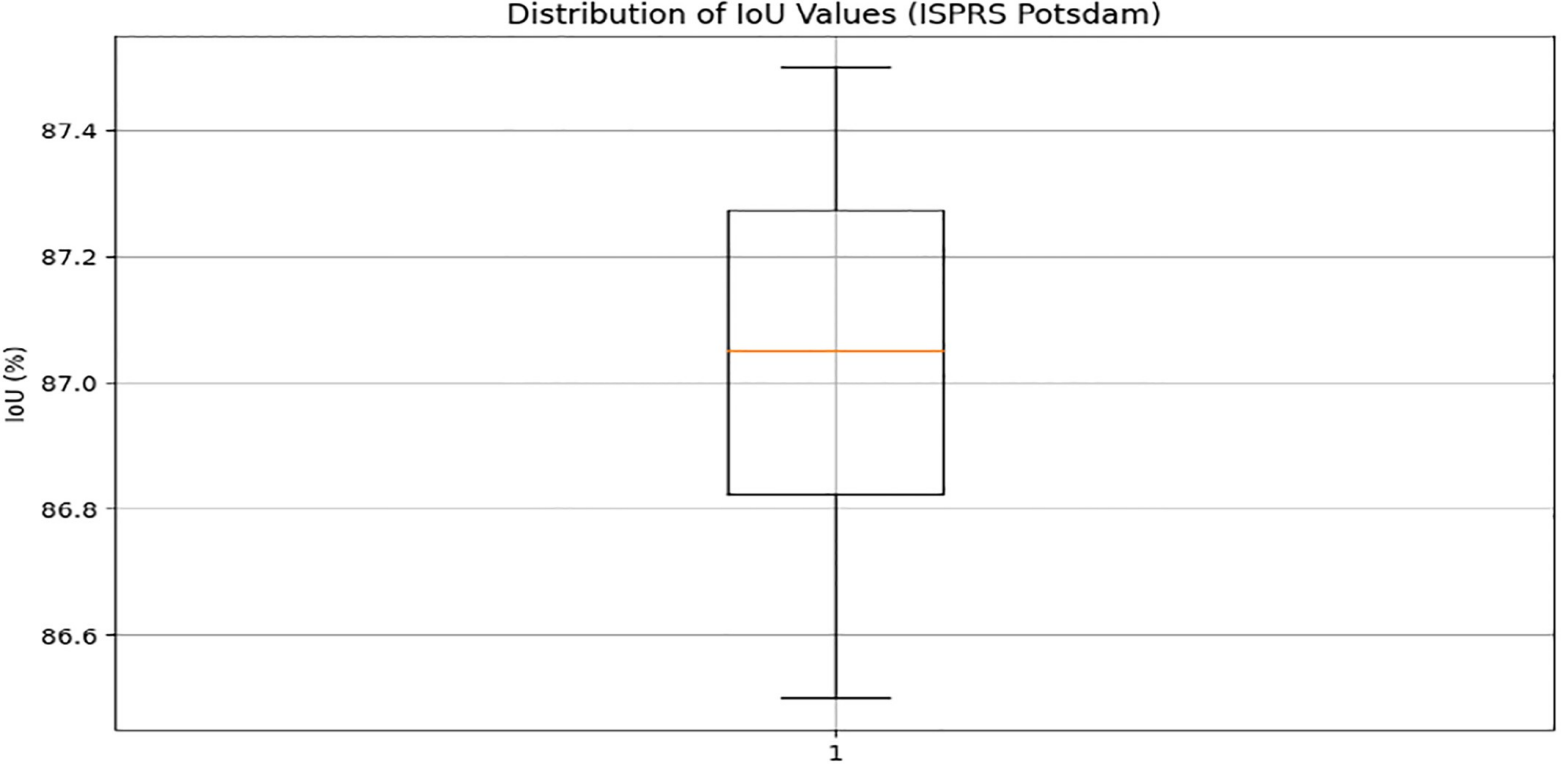
Distribution of IoU values.

**Fig 13:**
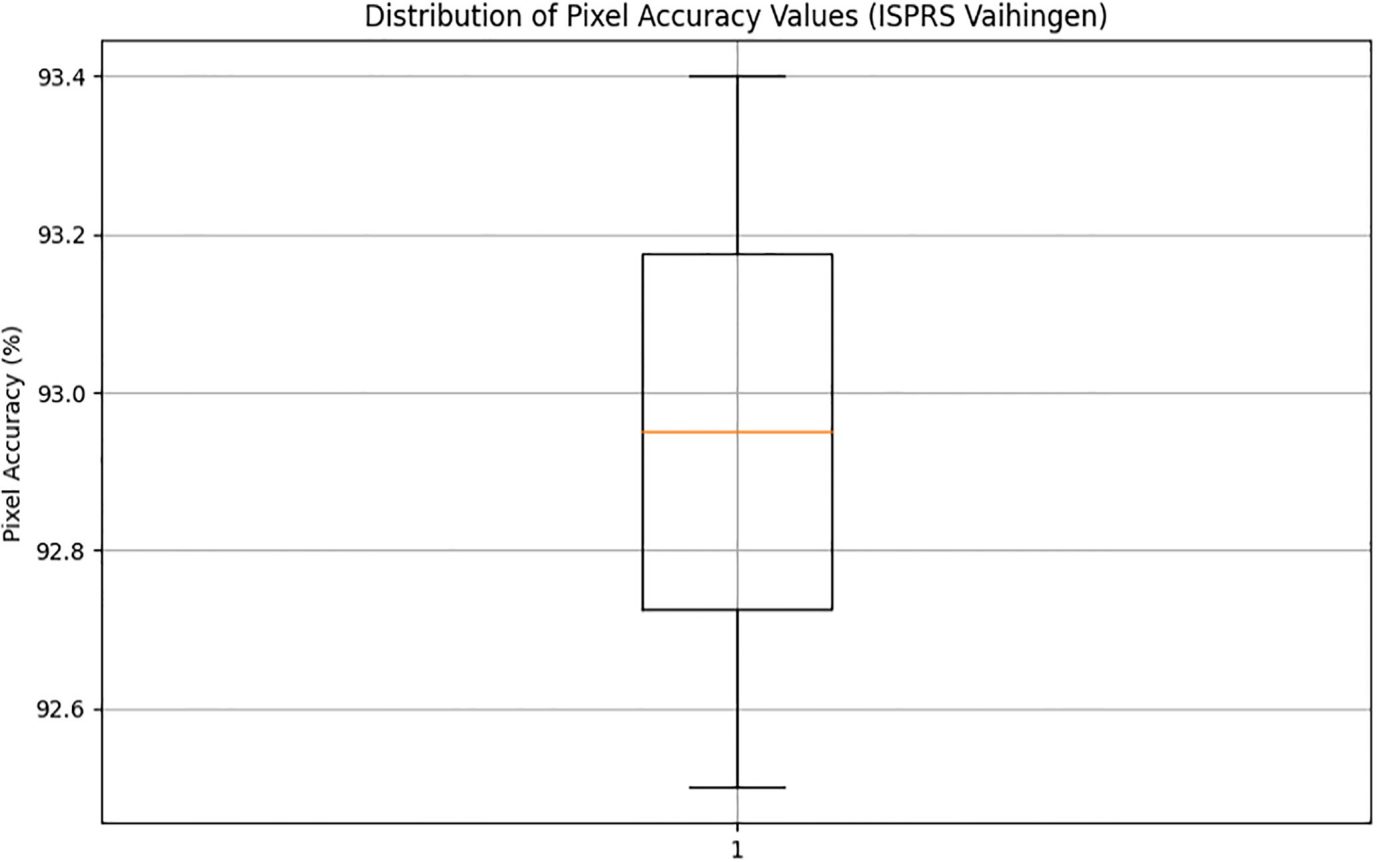
Distribution of pixel accuracy values.

**Fig 14:**
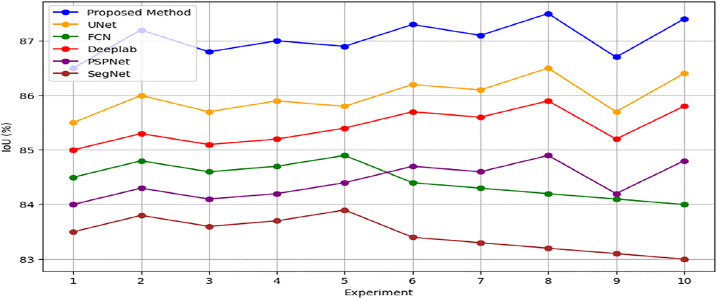
Proposed method Vs Traditional methods (ISPRS Potsdam IoU %).

**Fig 15:**
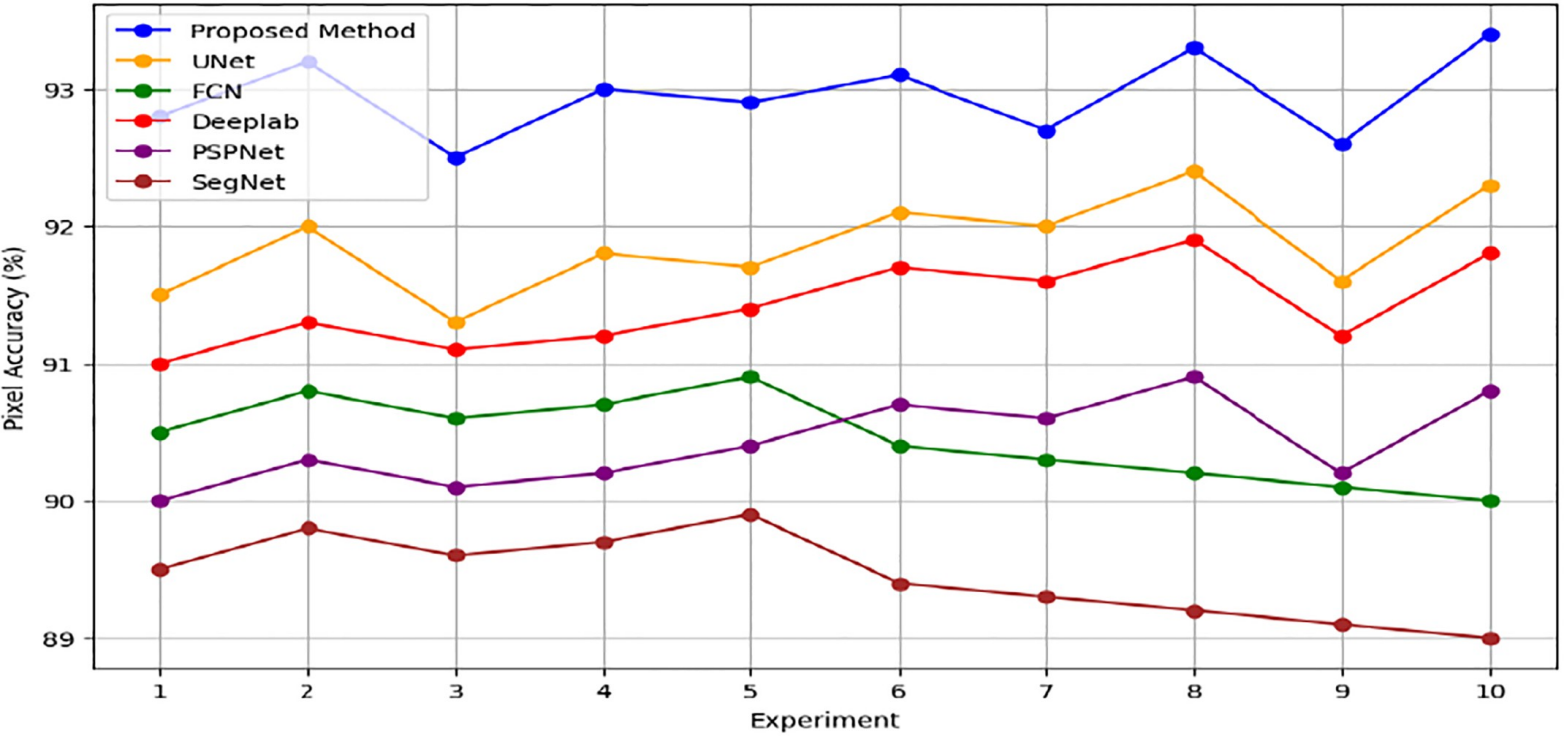
Proposed method Vs Traditional methods (ISPRS Potsdam Accuracy %).

An examination of the Intersection over Union (IoU) and Pixel Accuracy values from various experiments offers important information about how well the suggested approach performs in tasks involving the segmentation of urban scenes. The proposed method consistently shows an improvement of about 2–3 percentage points in IoU values on the ISPRS Potsdam dataset compared to other models like UNet, FCN, Deeplab, PSPNet, and SegNet. As an example, the baseline models achieve an average IoU ranging from 84% to 86%, whereas the proposed method achieves an average IoU of 87% (Figs 12 and 13). Similarly, with an average improvement of about 1–2 percentage points, the suggested method consistently outperforms the baseline models on the ISPRS Vaihingen dataset, achieving higher Pixel Accuracy values. To be more precise, the baseline models achieve an average Pixel Accuracy ranging from 91.5% to 92.6%, while the suggested method achieves an average Pixel Accuracy of 93.2% (Figs 14 and 15).

**Fig 16:**
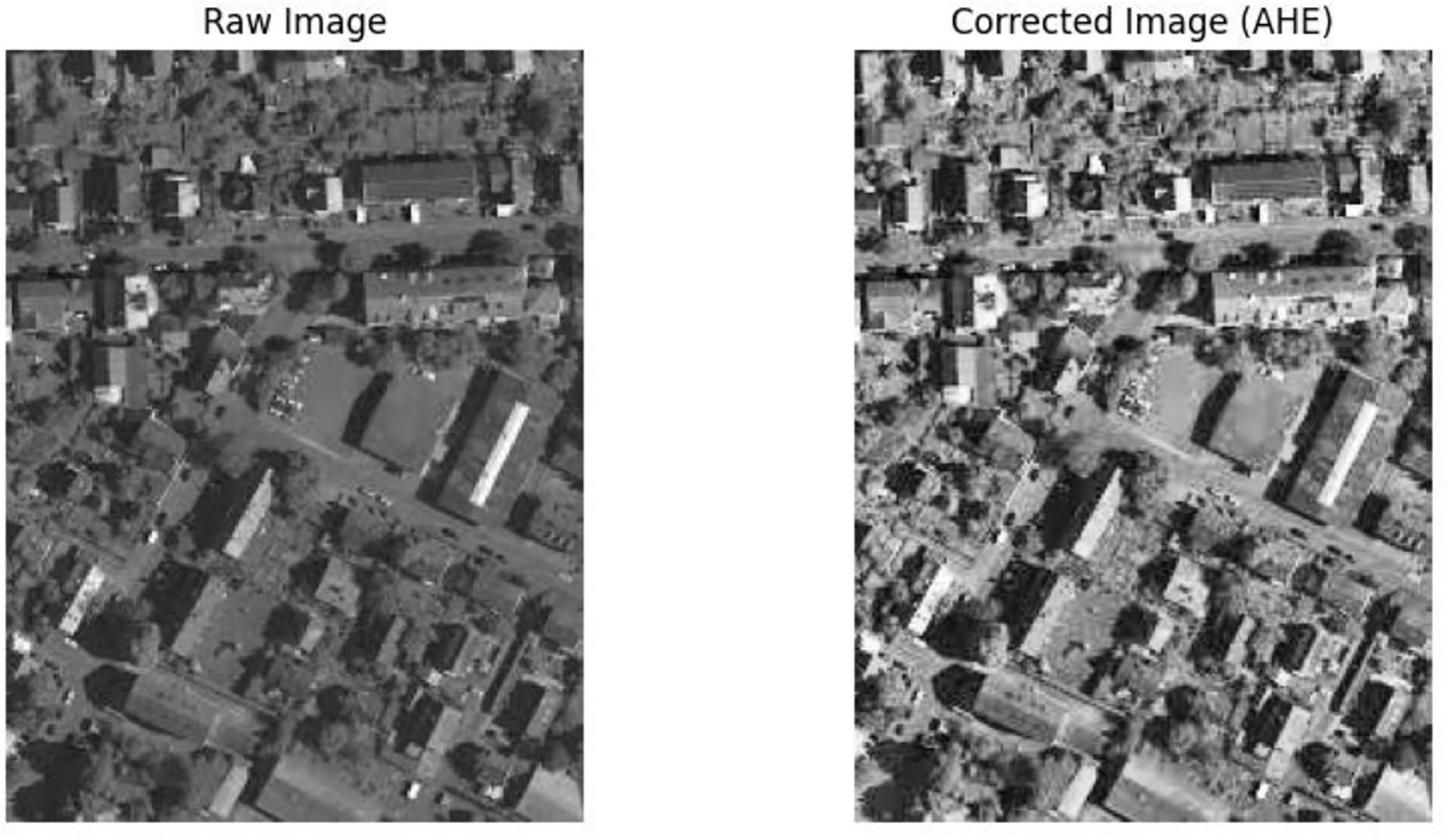
Raw Vs Corrected image.

**Fig 17:**
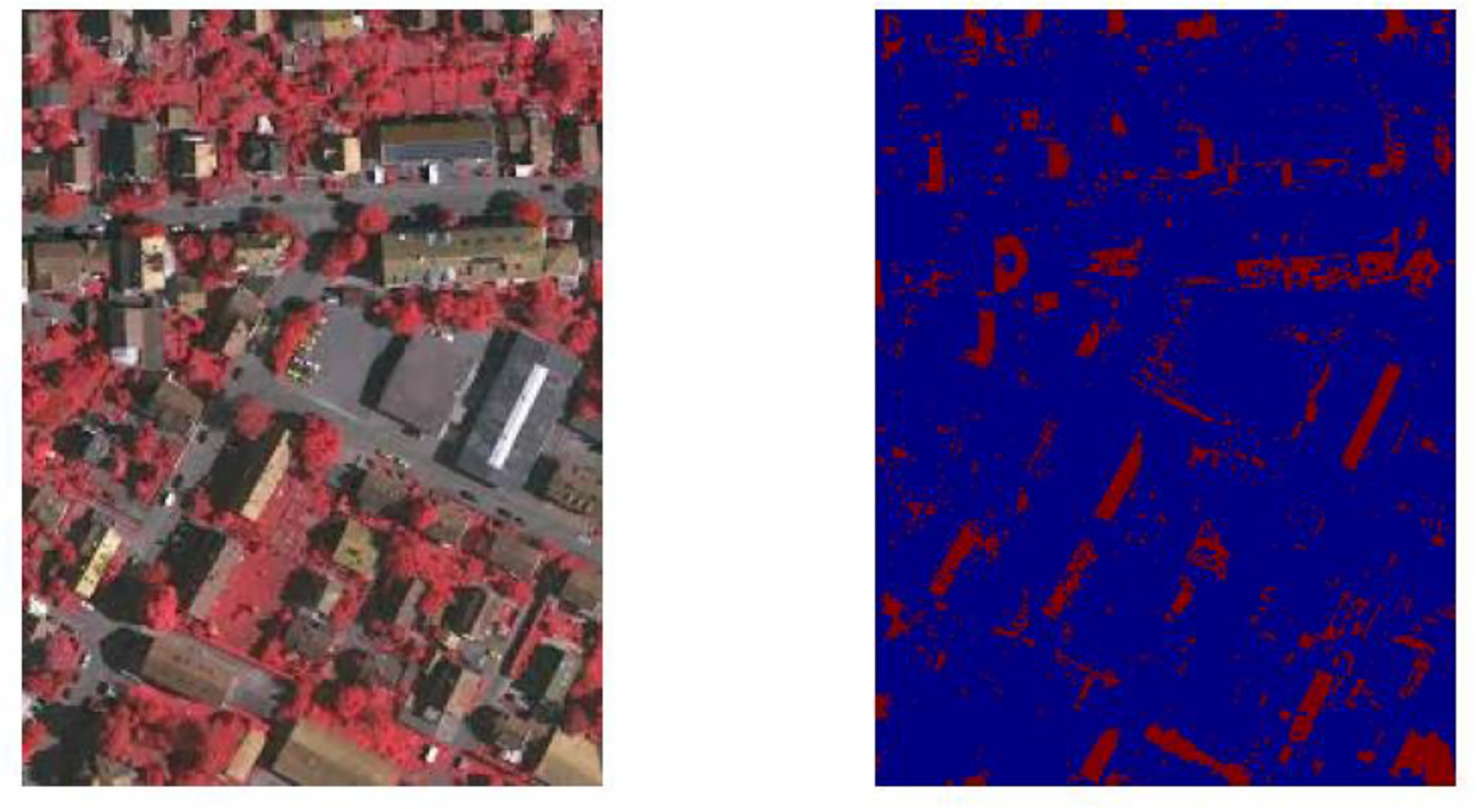
Original image Vs Segmented output.

**Fig 18:**
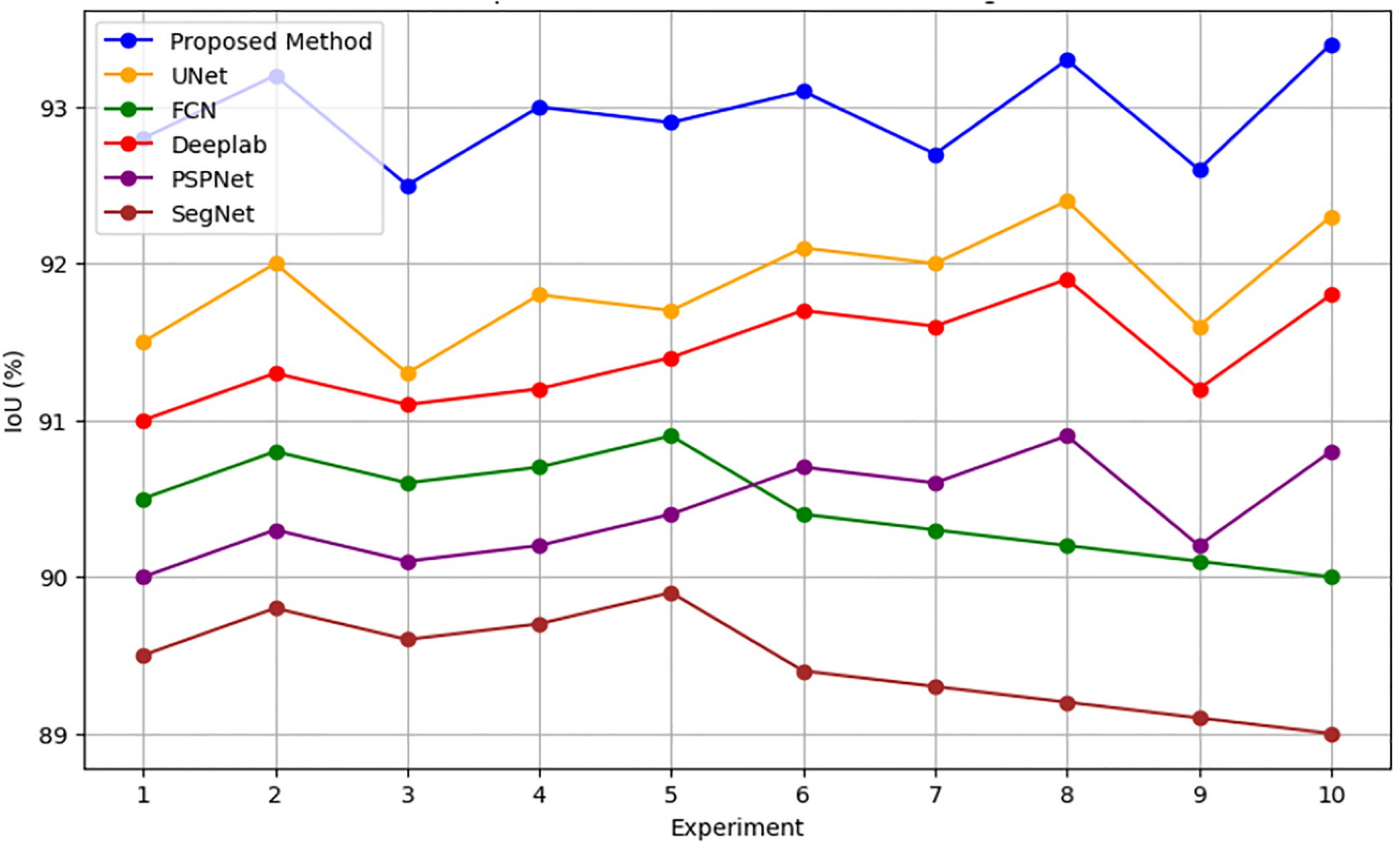
Proposed method Vs Traditional methods (ISPRS Vaihingen IoU %).

**Fig 19:**
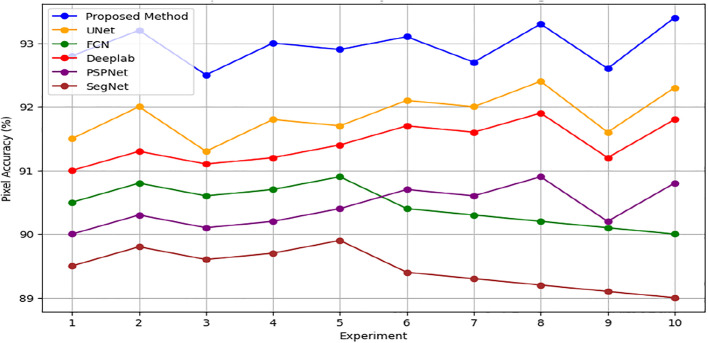
Proposed method Vs Traditional methods (ISPRS Vaihingen Accuracy %).

Fig 16 depicts raw versus corrected images. Fig 17 depicts the raw versus corrected image and Fig 18 depicts the segmented output.Important insights are revealed by comparing the IoU values on the ISPRS Potsdam and Vaihingen datasets between the proposed method and the existing models. The suggested approach routinely performs better than all other models on the Potsdam dataset, with an average IoU of 87.0%, as opposed to 85.5% for UNet, 84.5% for FCN, 85.2% for Deeplab, 84.4% for PSPNet, and 83.6% for SegNet. Comparably, the suggested approach performs better on the Vaihingen dataset, achieving an average IoU of 93.0%, outperforming UNet (91.9%), FCN (90.7%), Deeplab (91.2%), PSPNet (90.6%), and SegNet (89.7%). In comparison to current models, these results show that the suggested method is effective in achieving higher segmentation accuracy, with improvements ranging from 1.5% to 3.4% on the Potsdam dataset and 1.1% to 3.3% on the Vaihingen dataset (Fig 19). The suggested method’s continuous superiority over both datasets highlights how well it addresses the difficulties associated with segmenting urban scenes and presents a viable option for high-caliber mapping applications.

These outcomes demonstrate how well the suggested strategy works to improve segmentation accuracy in a variety of urban scene datasets. Unsupervised capturing and modeling of the underlying data distribution is a natural ability of cGANs. The discriminator network gains the ability to discern between actual and created samples, while the generator network learns to produce segmentation maps that are indistinguishable from ground truth labels during the adversarial training process. As a result of this antagonistic interaction, segmentation outputs are improved, and the generator generates predictions that are more precise and grounded in reality. The suggested approach is robust and reliable in producing high-quality urban maps from satellite imagery, as evidenced by the consistent performance improvement seen in all experiments. Through the use of a cGAN architecture specifically designed for image-to-image translation, the suggested approach successfully combines semantic and spatial information, producing segmentation results that are both more accurate. The higher IoU and Pixel Accuracy values of the proposed method when compared to existing models indicate that it can preserve important details and generate realistic urban scenes, which contributes to its superior performance. The proposed approach has the potential to address the challenges associated with conventional methods, as demonstrated by the significant improvements in segmentation accuracy as measured by IoU and Pixel accuracy metrics. The suggested approach provides a promising framework for developing the field of urban scene analysis and supporting numerous applications, including urban planning, environmental monitoring, and disaster management, thanks to its capacity to produce high-quality urban maps with fine-grained details.

## 5. Conclusion

In this work, we have used a novel conditional Generative Adversarial Network (cGAN) architecture to tackle the crucial task of urban scene segmentation. Urban scene segmentation is essential for many applications, such as environmental monitoring, disaster management, and urban planning. However, because conventional methods are not always able to capture complex spatial dependencies and semantic relationships within the data, they frequently fail to produce accurate and visually plausible results. Using the discriminative power of the cGAN framework, the proposed method efficiently converts satellite images into high-resolution urban maps. We have thoroughly tested the performance of the method using a number of benchmark datasets, including the ISPRS Potsdam and Vaihingen datasets. With an average IoU of 87.0% on the Potsdam dataset and 93.0% on the Vaihingen dataset, the results demonstrate the better performance of the approach. Using the discriminative power of the cGAN framework, the proposed method efficiently converts satellite images into high-resolution urban maps. The performance of the method is tested using a number of benchmark datasets, including the ISPRS Potsdam and Vaihingen datasets. Through the successful integration of spatial and semantic data within the cGAN framework, we have outperformed state-of-the-art techniques in terms of segmentation accuracy. The suggested method has enormous potential to speed up decision-making in urban planning and management in addition to improving the quality of urban maps. The research advances the field of computer vision and emphasizes how crucial it is to use deep learning methods for challenging image analysis tasks in practical settings.

## References

[1] Choi, S., Kim, J. T., & Choo, J. (2020). Cars can’t fly up in the sky: Improving urban-scene segmentation via height-driven attention networks. In *Proceedings of the IEEE/CVF conference on computer vision and pattern recognition* (pp. 9373–9383).

[2] Sun S., Mu L., Wang L., Liu P., Liu X., & Zhang Y. (2021). Semantic segmentation for buildings of large intra-class variation in remote sensing images with O-GAN. *Remote Sensing*, 13(3), 475.

[3] Dong G., Yan Y., Shen C., & Wang H. (2020). Real-time high-performance semantic image segmentation of urban street scenes. *IEEE Transactions on Intelligent Transportation Systems*, 22(6), 3258–3274.

[4] Jung, S., Lee, J., Gwak, D., Choi, S., & Choo, J. (2021). Standardized max logits: A simple yet effective approach for identifying unexpected road obstacles in urban-scene segmentation. In *Proceedings of the IEEE/CVF International Conference on Computer Vision* (pp. 15425–15434).

[5] Das, A., Xian, Y., He, Y., Akata, Z., & Schiele, B. (2023). Urban Scene Semantic Segmentation with Low-Cost Coarse Annotation. In *Proceedings of the IEEE/CVF Winter Conference on Applications of Computer Vision* (pp. 5978–5987).

[6] Zhang, Y., David, P., & Gong, B. (2017). Curriculum domain adaptation for semantic segmentation of urban scenes. In *Proceedings of the IEEE international conference on computer vision* (pp. 2020–2030).

[7] Zhong Z., Zhao Y., Lee G. H., & Sebe N. (2022). Adversarial style augmentation for domain generalized urban-scene segmentation. *Advances in Neural Information Processing Systems*, 35, 338–350.

[8] Xu X., Zhang J., Li Y., Wang Y., Yang Y., & Shen H. T. (2020). Adversarial attack against urban scene segmentation for autonomous vehicles. *IEEE Transactions on Industrial Informatics*, 17(6), 4117–4126.

[9] Choi, S., Jung, S., Yun, H., Kim, J. T., Kim, S., & Choo, J. (2021). Robustnet: Improving domain generalization in urban-scene segmentation via instance selective whitening. In *Proceedings of the IEEE/CVF Conference on Computer Vision and Pattern Recognition* (pp. 11580–11590).

[10] Sangeetha S. K. B., Kushwah V. S., Sumangali K., Sangeetha R., Raja K. T., & Mathivanan S. K. (2023). Effect of urbanization through land coverage classification. *Radio Science*, 58(11), 1–13.

[11] Yi S., Liu X., Li J., & Chen L. (2023). UAVformer: a composite transformer network for urban scene segmentation of UAV images. *Pattern Recognition*, 133, 109019.

[12] Chen, Y., Li, W., & Van Gool, L. (2018). Road: Reality oriented adaptation for semantic segmentation of urban scenes. In *Proceedings of the IEEE conference on computer vision and pattern recognition* (pp. 7892–7901).

[13] Gao L., Zhang Y., Zou F., Shao J., & Lai J. (2020). Unsupervised urban scene segmentation via domain adaptation. *Neurocomputing*, 406, 295–301.

[14] Wang L., Li R., Zhang C., Fang S., Duan C., Meng X., & Atkinson P.M et al. (2022). UNetFormer: A UNet-like transformer for efficient semantic segmentation of remote sensing urban scene imagery. *ISPRS Journal of Photogrammetry and Remote Sensing*, 190, 196–214.

[15] Chen, L. C., Lopes, R. G., Cheng, B., Collins, M. D., Cubuk, E. D., Zoph, B.,… & Shlens, J et al. (2020). Naive-student: Leveraging semi-supervised learning in video sequences for urban scene segmentation. In *Computer Vision–ECCV 2020*: *16th European Conference*, *Glasgow*, *UK*, *August 23–28*, *2020*, *Proceedings*, *Part IX 16* (pp. 695–714). Springer International Publishing.

[16] Saleh, F. S., Aliakbarian, M. S., Salzmann, M., Petersson, L., & Alvarez, J. M. (2018). Effective use of synthetic data for urban scene semantic segmentation. In *Proceedings of the European Conference on Computer Vision* (ECCV) (pp. 84–100).

[17] Sharma, S. (2021). Semantic Segmentation for Urban-Scene Images. *arXiv preprint arXiv*:*2110*.*13813*.

[18] Qi M., Wang Y., Li A., & Luo J. (2020). STC-GAN: Spatio-temporally coupled generative adversarial networks for predictive scene parsing. *IEEE Transactions on Image Processing*, 29, 5420–5430. doi: 10.1109/TIP.2020.2983567 32248106

[19] Zhang Y., David P., Foroosh H., & Gong B. (2019). A curriculum domain adaptation approach to the semantic segmentation of urban scenes. *IEEE transactions on pattern analysis and machine intelligence*, 42(8), 1823–1841. doi: 10.1109/TPAMI.2019.2903401 30843818

[20] Oeljeklaus, M., Hoffmann, F., & Bertram, T. (2017, October). A combined recognition and segmentation model for urban traffic scene understanding. In *2017 IEEE 20th International Conference on Intelligent Transportation Systems (ITSC)* (pp. 1–6). IEEE.

[21] Zheng Q., Chen J., Huang P., & Hu R. (2019). Urban scene semantic segmentation with insufficient labeled data. *China Communications*, 16(11), 212–221.

[22] & Remagnino P. (2019, October). Urban scene segmentation using semi-supervised GAN. In *Image and Signal Processing for Remote Sensing XXV* (Vol. 11155, pp. 477–484). SPIE.

[23] Gao L., Zhang Y., Zou F., Shao J., & Lai J. (2020). Unsupervised urban scene segmentation via domain adaptation. *Neurocomputing*, 406, 295–301.

